# Bacteriological Technique

**Published:** 1903-02

**Authors:** 


BACTERIOLOGICAL TECHNIQUE. A Laboratory Guide for the Medical, Dental, and Technical Student. By J. W. H. EYRE, M. D., F. R. S., Edin., Bacteriologist to Guy's Hospital, and Lecturer on Bacteriology at the Medical and Dental Schools, London. Octavo of 375 pages, with 170 illustrations. Philadelphia and London: W. B. Saunders & Co. 1902. [Cloth, $2.50 net.]

Dr. Eyre started out to make a book which would be of some use to the student who had a smattering of bacteriology and who wanted to know more, especially of that portion of the science which relates to technique and recognition of all the essential little details of bacteriology, and he has admirably succeeded. And one of the reasons for his success is his free use of illustrations. He is a believer in good pictures and those which are employed are admirable in every sense; they are artistic and eloquently useful. They have other needs than the filling up of space. A studied effort has been made to teach students how to fit up and adapt apparatus for daily use and so carefully has this been carried out that there can be little excuse for failure in technique.

The contents of this really valuable book shows how complete it all is. Nothing which could possibly be of the slightest use in the way of suggestion has been overlooked, nothing is taken for granted. Glassware is first taken up and described fully and the student is even taught how to clean and keep it clean. Sterilisation agents and methods follow, and then comes a most lucid and understandable chapter on the microscope, with microscopic examinations, included in which are descriptions of apparatus, reagents and their preparation, and the staining methods. This gives an idea of the thoroughness of the book and the great care given to detail.

Included in the work are these general chapter-headings: Methods of determining bacteria in tissue; methods of cultivation; isolation and identification; experimental inoculation of animals and post-mortem examinations; the study of the pathogenic bacteria, and bacteriologic analyses, all of which goes to make up a very complete volume. There are 170 illustrations and everyone has a distinct value in itself for each has a mission to fulfill and it achieves the object for which it was used.

